# Development of the set of scales to assess the job satisfaction among physicians in Peru: validity and reliability assessment

**DOI:** 10.1186/s12889-021-11964-6

**Published:** 2021-10-24

**Authors:** David Villarreal-Zegarra, Roberto Torres-Puente, Ronald Castillo-Blanco, Baltica Cabieses, Luciana Bellido-Boza, Edward Mezones-Holguin

**Affiliations:** 1 Universidad César Vallejo, Escuela de Medicina, Trujillo, Peru; 2 Instituto Peruano de Orientación Psicológica, Lima, Peru; 3 Universidad del Pacífico, Gestión del Aprendizaje y Aseguramiento de la Calidad, Lima, Peru; 4 Universidad del Desarrollo, Facultad de Medicina Clínica Alemana, Instituto de Ciencias e Innovación en Medicina (ICIM), Santiago de Chile, Chile; 5 Intendencia de Investigación y Desarrollo Superintendencia Nacional de Salud, Lima, Peru; 6 Universidad Peruana de Ciencias Aplicadas, Facultad de Ciencias de la Salud, Lima, Peru; 7 Universidad San Ignacio de Loyola, Centro de Excelencia en Investigaciones Económicas y Sociales en Salud, Lima, Peru; 8 Epi-gnosis Solutions, Piura, Peru

**Keywords:** Job satisfaction, Physicians, Psychometrics, Health services research, Peru

## Abstract

**Background:**

To assess the validity and reliability of the set of scales (general professional activity, health services management, and working conditions) on the different areas of job satisfaction in Peruvian physicians based on the data from the National Survey of Satisfaction of Users in Health (ENSUSALUD).

**Method:**

We carried out a psychometric study based on the secondary data analysis of Questionnaire 2 of ENSUSALUD-2016. Participants were selected from a two-stage stratified national probability representative sampling by political region. Validity was assessed by exploratory and confirmatory factor analyses, and measurement invariance analysis. We assessed the reliability using internal consistency coefficients (alpha and omega). The set of scales were composed of items related to three different areas of job satisfaction: 1) satisfaction with general professional activity, 2) satisfaction with the health services management, and 3) satisfaction with the working conditions of the health center.

**Results:**

We included 2137 participants in the analysis. The general professional activity scale with six items (Comparative Fit Index, CFI = 0.946; Root Mean Square Error of Approximation, RMSEA = 0.071; Standardized Root Mean Square Residual, SRMR = 0.035), the health services management scale with eight items (CFI) = 0.972; RMSEA = 0.081; SRMR = 0.028), showed good measurement properties for the one-dimensional model. The working conditions scale with eight items for individual conditions and three items for infrastructural conditions (CFI = 0.914; RMSEA = 0.080; SRMR = 0.055) presented adequate measurement properties with a two-dimensional model. The invariance analysis showed that comparisons between sex, age, civil status, medical speciality, working in other institutions, work-related illness, chronic disease, and time working in the healthcare center. All scales had adequate internal consistency (ω and α between 0.70 and 0.90).

**Conclusions:**

The set of scales has a solid factorial structure and measurement invariance, making it possible for group comparison. The study achieved stability in the scores as they showed adequate internal consistency coefficients. Based on our findings, these instruments are suitable for measuring job satisfaction among outpatient physicians throughout Peru, as our data is representative of the country level.

**Supplementary Information:**

The online version contains supplementary material available at 10.1186/s12889-021-11964-6.

## Background

Job satisfaction is an emotional state or attitude toward a job based on positive or negative experiences and worker values or expectations [1]. International evidence suggests that the health workers with higher job satisfaction improve employee performance and patients’ perceptions of care quality [2, 3]. Thus, job satisfaction is a critical concern to help improve health policies, since it can positively affect the health workers’ performance and patients’ satisfaction [3]. However, for low levels of job satisfaction among health workers, detrimental results appear, such as burnout, employee turnover, job change, and poor working performance [4, 5]. These poor functioning and quality outcomes worsen accountability and resilience of healthcare systems, contributing to pervasive health gaps between and within socio-economic groups [4, 5]. Therefore, evaluating health workers job satisfaction, including the physician who often leads healthcare teams, is a significant dimension to consider in the global public health agenda.

There is an urgent need to assess physicians’ job satisfaction in low-income and middle-income countries since they struggle with complex labor dynamics more often than their peers who work in high-income developed countries [6]. In addition, job dissatisfaction may lead to the migration of health workers in many developing countries overseas, causing specialists shortages [7–9]. Although job satisfaction in physicians is relevant, its assessment is highly complex as it requires evaluating various factors and dimensions of the work environment. Job satisfaction can be associated with the doctor-patient relationship, workload, relationship with colleagues, financial conditions, and autonomy in clinical decision-making [10, 11]. These factors can be used to develop measurement tools in complex scenarios such as the workplace. Notably, there are many scales for assessing job satisfaction, but many of these instruments have not been adapted to low-income and medium-income contexts, let alone consider each of these healthcare systems [11, 12]. Thus, it is necessary to have instruments that are contextualized to the characteristics of each healthcare system.

Peru is a middle-income country in Latin America that has suffered historical and structural difficulties and deficiencies in the public health arena, including financial crises. Due to these limitations, the job satisfaction of the healthcare personnel has received less attention [13]. Peru has an underdeveloped healthcare system, lacks sufficient human resources and financial support, which contributes directly to the reproduction of inequities in healthcare [14, 15]. Hence, in this country, the lack of a robust measure of physician job satisfaction which were valid and reliable, could limit diagnosis and follow up on this issue, impact health policy planning, and human resource sustainability. In 2016, the National Health Authority (Superintendencia Nacional de Salud–SUSALUD, from the Spanish acronym) carried out a national survey called National Survey of Satisfaction of Users in Health (“Encuesta Nacional de Satisfacción de Usuarios en Salud”–ENSUSALUD, from the Spanish acronym) to evaluate the user satisfaction of universal health insurance on six different populations in Peruvian Health System. One section was performed by doctors working in healthcare centers. ENSUSALUD included questions related to the job satisfaction of these professionals; nonetheless, no formal analysis was carried out to assess the validity and reliability of these instruments.

Consequently, our objective was to evaluate the validity and reliability of the set of scales (general professional activity, health services management, and working conditions) on different areas of job satisfaction in Peruvian physicians based on the data from ENSUSALUD. The results could contribute to measure the improvement concerning physicians’ job satisfaction in Peru.

## Methods

### Design and data source

We carried out a psychometric study based on the secondary data analysis of Questionnaire 2 of ENSUSALUD-2016. Doctors and nurses filled out this questionnaire in healthcare centres; we performed our analysis explicitly on physicians data. The database is publicly available on the web (http://portal.susalud.gob.pe/blog/base-de-datos-2016/).

ENSUSALUD 2016 was developed by the Peruvian National Institute of Statistics (INEI, from the Spanish acronym) in collaboration with SUSALUD. This survey was performed in 185 healthcare centers in all 25 regions of Peru [16]. Professionals who had worked for a minimum of 12 months in healthcare centres, and public or private sector were included: Ministry of Health (MINSA, from the Spanish acronym), Social Security (EsSalud, from the Spanish acronym), armed forces and police health services (AFPHS), and private subsector.

### Participants

Participants were selected from a complex two-stage stratified national probability representative sampling by political region. The primary sampling unit were the healthcare centres, and the secondary sampling unit were professionals. Physicians over 65 years were excluded (retirement age in Peru). We included only participants with complete data on all satisfaction scales.

### Generation and development

In 2014 and 2015, before ENSUSALUD 2016, there were the first two attempts to develop a job satisfaction scale for healthcare workers in the country. The process of developing these instruments was two-folded:

#### First phase: ENSUSALUD 2014 and ENSUSALUD 2015

ENSUSALUD was developed in 2014 by SUSALUD. In that process, other institutions, including the MINSA, suggest topics to measure in the questionnaires of this survey. During the first half of 2014, a multidisciplinary technical team (from Health Services Quality Directorate of the MINSA, Research and Development Intendance of SUSALUD, and INEI), conducted an extensive review of the literature on working conditions in the health system and on the operational evaluation instruments previously used in the Peruvian Health System. From this, 53 preliminary scales to assess different aspects of health professionals’ work (physicians and nurses) with additional sociodemographic data [17]. Each preliminary scale had 1 to 22 items, and they were all included in the first version of ENSUSALUD 2014. The preliminary scales were groups of items based on instruments already designed or designed ad hoc to evaluate the Peruvian health system (in this case, the measurement properties have not been evaluated). Subsequently, in ENSUSALUD 2015, the same technical team used the 53 preliminary scales, added other specific scales, and modified the wording of some items based on the previous experience [18].

#### Second phase: ENSUSALUD 2016

In 2016, SUSALUD convened EsSalud, AFPHS, officials of the comprehensive health insurance, and four universities in Lima, Peru. They discussed the modifications to the existing questionnaires. The decision was to keep all the questions and items from the previous versions of job satisfaction, but with certain modifications. Then, they included 30 items in three groups (three preliminary scales) in questionnaire 2 of ENSUSALUD 2016. Two authors of our manuscript (LBB y EMH) participated in this process.

Likewise, we do not have access to the initial theoretical review conducted by the MINSA to formulate the scales in 2014. Nevertheless, the three scales fit the theoretical model: “social processing of information at work” [19, 20]. This model explains that job satisfaction is based on:

- individual perception and affective evaluation of the work situation (satisfaction scale on the general professional activity),
- the social context that provides information on the attitudes toward the environment (Health Services Management Satisfaction Scale), and
- the perception that the individual has the conditions that lead him/her to manifest certain behaviours (Satisfaction scale on the working conditions of the health centre).

### Procedures

The INEI (Peruvian National Institute of Statistics, a public entity) evaluator collected the individual data from the physicians at a healthcare centre by a personal interview. All data collection processes were constantly monitored through a network of supervisors and real-time cross-validation. The surveys were conducted using a tablet, and SUSALUD coordinated the authorizations with the healthcare centre authorities.

### Measuring instruments

The thirty-item job satisfaction questionnaire of ENSUSALUD evaluates different job-related aspects by three different scales: general professional activity (6 items), health services management (8 items), and working conditions of the health centres (16 items). Each of these items used Likert-type scale with five options (5 = very satisfied, 4 = satisfied; 3 = neither satisfied nor dissatisfied; 2 = dissatisfied; 1 = very dissatisfied). We presented the Spanish version (Additional file 1) and preliminary English version (Additional file 2). All items assessed in the ENSUSALUD are open access and can be found in the ENSUSALUD questionnaire 2 (http://portal.susalud.gob.pe/wp-content/uploads/archivo/encuesta-sat-nac/2016/Cuestionario%202%20-%20Profesionales%20medicos%20y%20enfermeria.pdf). At the moment, we did not found articles about these scales published in any indexed journal. The three scales were:

- *Satisfaction scale on general professional activity:*

To explore several general aspects of professional labor. The items evaluate the satisfaction of the doctor-patient relationship, achievements associated with the profession, work availability, perception of occupational risk, and expectations in meeting the patient’s needs. Within ENSUSALUD, the items in Spanish of this instrument are in question 82 with codes from c2p82_1 to c2p82_6 (see Additional file 1).

b) *Satisfaction scale on health services management:*

To assess the healthcare facility’s management team. The items included in this scale are satisfaction with resource management (economic and human), drug management, shift scheduling, and work capacity. In ENSUSALUD, the items of this instrument are in question 83 with codes from c2p83_1 to c2p83_8 (see Additional file 1).

iii) *Satisfaction scale on the working conditions of the health centers:*

To evaluate the working conditions perceived by the health professional. The scale indicators are satisfied with the possibility of promotion, health centers organization, workload, schedules, salary, opportunities, infrastructure and equipment, relationship with superiors, administrative procedures, and hygiene of the health centers. In ENSUSALUD, the items of this instrument are in question 81 with codes from c2p81_1 to c2p81_16 (see Additional file 1).

In addition, we included demographic, professional, and economic information in our analysis. Sex, age and marital status (living as a couple) were the demographic variables. We also evaluated professional information: speciality (yes, residency, or no), working in other healthcare centers (yes / no), self-reported work-related illness (yes / no), institution (Ministry of Health, EsSalud, armed forces and national police, or private clinics), and weekly time spent at work. In addition, self-reported monthly income was evaluated and categorized according to the minimum wage (less than four, four to ten, and more than ten). The minimum wage in 2016 was 750 Peruvian soles (PEN) or $222.5 (considered to be an exchange rate of 3.37 soles per US dollar).

### Statistical analysis

#### Descriptive analysis

We presented the general characteristics of the participants using weighted frequencies and percentages.

#### Exploratory factor analysis (EFA)

We analyzed a random subset from the total sample (split-half method) [21, 22]. We used polychoric matrices [23], and the estimator was weighted using least-squares means and variance adjusted (WLSMV) [24], since it best fitted with the ordinal nature of our items. We applied quartimin rotation, parallel analysis test and Kaiser analysis to evaluate the most appropriate number of dimensions [25]. We obtained different models and evaluated them to identify the best with measurement properties based on theoretical models that suggest that job satisfaction is a multidimensional construct. Before performing exploratory factorial analysis, we estimated the value of the Kaiser-Meyer-Olkin (KMO). This index of sample adequacy allows identifying whether there is enough power or sample size to perform the analysis. KMO values higher than 0.90 are adequate [23].

To evaluate the factor structures, we used three different criteria. First, items factor loadings should be equal to or greater than 0.40 [21]. Second, if a scale has more than one dimension, each dimension must have at least three items to be considered stable [26]. Third, if an item loads more than one dimension and their difference is lower than 0.020, it will be deleted. Moreover, the difference in loadings, equal to or greater than 0.20, implies the item’s inclusion in the dimension with the highest factor load [21].

#### Confirmatory factor analysis (CFA)

For CFA, we evaluated the models previously obtained in the exploratory factor analysis. The estimator used were WLSMV [24] and polychoric matrices [23]. We used different goodness-of-fit indices to evaluate the CFA since none by itself would allow a complete evaluation of factorial complexity. We used the Comparative Fit Index (CFI) and the Tucker-Lewis Index, both with the optimal value of ≥0.95. In addition, the Standardized Root Mean Square Residual (SRMR) and Root Mean Square Error of Approximation (RMSEA) with a confidence interval of 90%, both with adequate values if < 0.08 [27]. If the models presented two or more dimensions, the latent correlation between both dimensions was evaluated, since if the latent relationship was very high, the dimensions could be overlapping and be evaluating the same construct. We established a cut-off point to define that the latent dimensions were differentiated if the latent correlation was less than 0.80 [24].

#### Measurement invariance

We performed invariance analysis to evaluate whether different groups had the equivalent understanding of the construct assessed; if the groups were equivalent to each other, they are defined as invariant, and therefore comparisons can be made between them. The grouped relevant variables were: sex, age group, marital status, medical speciality, work in other institutions, individual income per month, self-reported work-related illness, and self-reported chronic illness. The invariance analysis focuses on performing progressive restrictions on the different categories of the groups to compare the extent to which they are equivalent [28, 29]. The change in CFI (ΔCFI) less than 0.01 was used as the main criterion to define that the comparison between models with more restrictions versus models with fewer restrictions (configural, thresholds, metric, and scalar) [29]. Invariance was considered to exist between the evaluated groups when the ΔCFI was less than 0.01. We preferred ΔCFI over χ^2^ comparisons since it is not sensitive to big sample sizes [28, 29].

#### Reliability

We evaluated reliability by internal consistency method, taking the optimal value of McDonald’s omega coefficient (ω) and alpha coefficient (α). In both cases, values > 0.70 were categorized as appropriate [30–33].

#### Satisfaction levels

Exploratory based on the ENSUSALUD manual, we categorized it as satisfied when the answer was very satisfied or satisfied (response option 5 or 4).

We performed all analyses considering the complex characteristics of the sampling strategy (complex multi-stage sampling) in R Studio®, specifically with the packages “lavaan” [34], “lavaan.survey” [35], “semTools” [36], and “semPlot” [37].

### Ethics

Since our study is a secondary analysis using public open-access databases without access to personal data, we did not submit the protocol to Ethics Committee. We used only data collected by SUSALUD. During data collection conducted by INEI, they followed all ethical guidelines of ENSUSALUD 2016, including an electronic record of verbally informed consent of all participants by tablet.

## Results

### Descriptive analysis

The SUSALUD database used for this research has 98% response rate. The database initially has 2216 physicians; however, we removed 79 observations because they did not meet the inclusion criteria. Therefore, 2137 physicians were included, which represent 96.43% of the original database. In this study, we included only participants with complete data in all satisfaction scales. The majority of the participants were men (69.0%), living as a couple (married or cohabiting), more than half with speciality, 65% with monthly income of four to ten minimum wages ($890 to $2225), one in four with work-related illness, and one in three self-reported a chronic disease. Average age was 44.7 years (SD = 10.8) and the average time worked in the organization was 9.4 years (SD = 9.2). We presented the general characteristics of physicians in Table 1.

**Table 1:** Description of the sample of physicians included in the analysis (ENSUSALUD 2016) (*n* = 2137)

		*n*	%
Sex	Men	1598	69.0%
	Women	539	31.0%
Age	23 to 29	145	7.7%
	30 to 39	664	31.8%
	40 to 49	595	25.8%
	50 to 65	733	34.8%
Civil status	Living with a couple (married or cohabiting)	1483	64.4%
	Living without a partner (single, divorced, separated and widowed)	654	35.7%
With specialty	Yes	1243	52.1%
	No, in process	344	12.0%
	No	550	36.0%
Working in other workplace	Yes	932	41.5%
	No	1205	58.5%
Monthly income	< 4 minimun wages	70	4.2%
	4–10 minimum wages	1421	65.0%
	More to ten minimum wages	610	29.3%
	No report	36	1.5%
Work-related illness	Yes	487	23.0%
	No	1650	77.0%
Chronic Disease	Yes	563	30.3%
	No	1575	69.7%
Institution	Ministry of Health	979	43.3%
	Social Security (EsSalud)	999	37.4%
	Armed forces and Police Services	33	8.3%
	Private subsector	126	14.0%
Time working in the health center	Two years or less	691	36.5%
	3 to 5 years	405	19.6%
	6 to 10 years	296	12.5%
	11 years or more	745	31.4%

Monthly income = Less than four minimum wages (≤$890), four to ten minimum wages ($890 to $2225) or more than ten minimum wages (≥$2225)

### Exploratory factor analysis

#### Satisfaction scale on general professional activity

The KMO value was greater than 0.90, suggesting an adequate sample size to perform the exploratory factor analysis. The parallel analysis identified two possible dimensions, and Kaiser’s analysis identified a single dimension. Due to this heterogeneity in our findings, we assessed one and two-dimension models at this stage. The one-dimensional model showed adequate factor loadings (λ > 0.40; see Table 2), but the two-dimension model did not meet the criteria of having at least three items for each dimension. Therefore, we did not consider this two-dimension model for additional analyses.

**Table 2:** Exploratory Factor Analysis on three satisfaction scales evaluated. (*n* = 2137)

		One-factor model		Two-factor model			Three-factor model		
Scales	Items	F1		F1	F2		F1	F2	F3
Satisfaction scale on general professional activity	c2p82_1	0.449		−0.667	0.449		–	–	–
	c2p82_2	0.521		−0.617	0.521		–	–	–
	c2p82_3	0.506		–	0.506		–	–	–
	c2p82_4	0.497		–	0.497		–	–	–
	c2p82_5	0.620		–	0.620		–	–	–
	c2p82_6	0.537		–	0.537		–	–	–
Health Services Management Satisfaction Scale	c2p83_1	0.773		0.851	–		0.850	–	–
	c2p83_2	0.769		0.853	–		0.806	–	–
	c2p83_3	0.848		0.762	–		0.773	–	–
	c2p83_4	0.812		0.715	–		0.759	–	–
	c2p83_5	0.568		–	0.508		–	0.529	–
	c2p83_6	0.690		–	0.819		–	–	0.421
	c2p83_7	0.663		–	0.442		–	–	0.762
	c2p83_8	0.805		–	0.451		–	–	0.446
Satisfaction scale on the working conditions of the health center	c2p81_1	–		0.554	–		–	0.771	–
	c2p81_2	–		0.400^a^	0.423^a^		–	0.499	–
	c2p81_3	–		0.642	–		–	0.566	–
	c2p81_4	–		0.550	–		–	–	–
	c2p81_5	–		0.660	–		0.717	–	–
	c2p81_6	–		0.588	–		–	0.616	–
	c2p81_7	–		–	–		–	–	–
	c2p81_8	–		0.569	–		0.648	–	–
	c2p81_9	–		0.444	–		–	–	–
	c2p81_10	–		–	–		–	0.472	–
	c2p81_11	–		–	0.844		–	–	0.816
	c2p81_12	–		–	0.803		–	–	0.792
	c2p81_13	–		0.518	–		–	0.459	–
	c2p81_14	–		–	0.701		–	–	0.739
	c2p81_15	–		–	–		–	–	0.416
	c2p81_16	–		–	–		–	–	–

Note: Only factor loads between 0.400 and 1.000 are shown. ^a^ The difference of the factorial loading is lower of 0.200 between the factors

#### Health services management satisfaction scale

KMO value was higher than 0.90, suggesting a good proportion of variance among variables that might be a common variance. The parallel analysis identified three possible dimensions, and Kaiser’s analysis identified two dimensions. Due to the heterogeneity, we evaluated one, two and three dimensions models. The one-dimensional and two-dimensional models presented adequate factor loadings for physicians (λ > 0.40) and met the condition of having at least three items in each dimension (see Table 2). On the other hand, the structure of the three-dimensional model was very heterogeneous. The dimensions were not stable since they had very few items (less than three items per dimension). Therefore, we did not consider this model in subsequent analyses.

#### Satisfaction scale on the working conditions of health centers

KMO value was greater than 0.90, which suggests an adequate sample size to perform the exploratory factor analysis. The parallel analysis identified a two-dimensional model, and Kaiser’s analysis identified a three-dimensional model. Consequently, two and three-dimensional models were evaluated (see Table 2). In the model with two dimensions, the item “order in the health service and labor organization” (variable c2p81_2 in the dataset) presented a factor complexity since there was no marked difference between factor loadings in the first and second dimensions. Consequently, this item was removed from the analysis. In addition, the items on satisfaction about the hours or salary received (c2p81_7), training opportunities (c2p81_10), filling out the medical records (c2p81_15), and respect for the patient (c2p81_16), presented very low factor loadings, thus, those were eliminated from subsequent analyses. Moreover, we found that the first dimension was unstable (very few items) in the three-dimensional model, so this model was also eliminated. After excluding the five problematic items identified during this analysis, we considered only the two-dimensional model for further analysis (c2p81_2, c2p81_7, c2p81_10, c2p81_15, and c2p81_16).

### Confirmatory factor analysis

#### Satisfaction scale on general professional activity

The one-dimensional model evaluated, achieved the adequate goodness-of-fit indices (see Table 3), so the six items on this scale were added up into the overall score.

**Table 3:** Confirmatory Factor Analysis of the three scales evaluated (*n* = 2137)

Scales	Model	X^2^ (df)	CFI	TLI	RMSEA [90% CI]	SRMR	φ F1-F2
Satisfaction scale on general professional activity	One-factor	29.170 (9)	0.946	0.909	0.071 [0.043–0.100]	0.035	–
Health Services Management Satisfaction Scale	One-factor	75.319 (20)	0.972	0.961	0.081 [0.062–0.101]	0.028	–
	Two-factor	45.774 (19)	0.986	0.980	0.059 [0.037–0.080]	0.023	0.927
Satisfaction scale on the working conditions of the health center	Two-factor	125.047 (43)	0.914	0.890	0.080 [0.064–0.097]	0.055	0.506

X^2^ = Chi-squared. *df* Degrees of freedom. *CFI* Comparative fit index. *TLI* Tucker-Lewis index. *RMSEA* Root mean square error of approximation. *SRMR* Standardized root mean square residual. *φ* Latent relationship between dimensions F1 and F2

#### Health services management satisfaction scale

The one-dimensional model and the two-dimensional model had adequate goodness-of-fit indices. However, the two-dimensional model has an extremely high latent correlation (greater than 0.80), suggests that its dimensions might overlap (see Table 3). Hence, the best model for this scale was the one-dimensional one with eight items.

#### Satisfaction scale on the working conditions of the health center

**Fig. 1:**
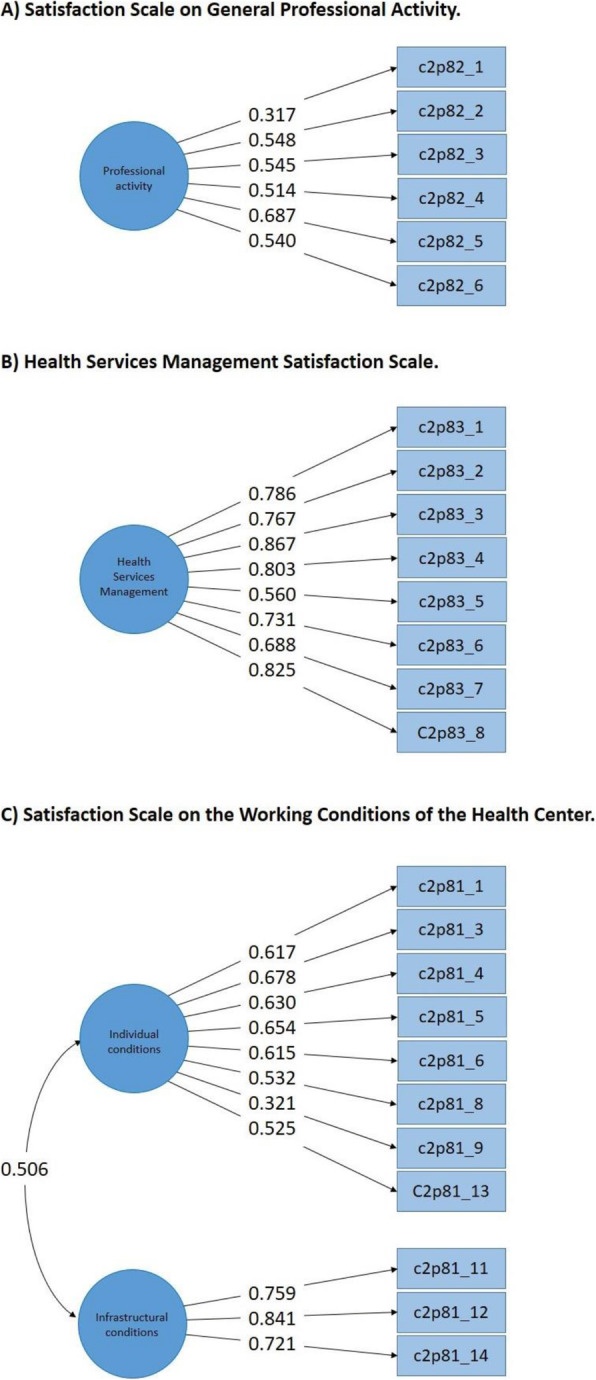
Factorial structure of the three scales evaluated

The two-dimensional model consisting of eleven items showed adequate goodness-of-fit indices, and the latent correlation between the two dimensions was also within the appropriate values (less than 0.80, see Table 3). The first dimension was composed of eight items related to the satisfaction of physician’s working conditions (i.e., workload, hours, salary), and the second dimension had three items related to structural working conditions (i.e., infrastructure, equipment). From this analysis, this model presented adequate validity based on its internal structure, therefore, was considered for further analysis (see Fig. 1).

### Measurement invariance

#### Satisfaction scale on general professional activity

Invariance was reached between marital status, chronic disease, and work-related disease. Therefore, we executed comparisons between these groups. We observed violations of invariance between sex (men and women), people working in other institutions, and time working in the primary institution. Thus, we did not perform comparisons between these variables (See Table 4). Finally, since it was impossible to evaluate the invariance according to the type of organization, monthly income, speciality, or participant’s age, necessary assumptions for such analysis were not fulfilled.

**Table 4:** Measurement invariance between groups for the three scales evaluated (*n* = 2137)

									DIFFTEST		
Scale	Group	Invariance	X^2^-Robust	*df*	CFI	RMSEA	SRMR	ΔCFI	Value	*df*	*p*
Satisfaction scale on general professional activity	Sex ^a^	Configural	659.1	18	0.920	0.120	0.062	–	–	–	–
		Thresholds	733.4	30	0.913	0.097	0.062	−0.008	23.1	12	0.027
		Metrict	689.4	35	0.919	0.087	0.063	0.006	9.8	5	0.081
		Scalar	790.4	40	0.907	0.087	0.063	−0.012	71.3	5	0.000
	Civil status	Configural	655.0	18	0.920	0.119	0.062	–	–	–	–
		Thresholds	716.1	30	0.913	0.096	0.062	−0.006	14.9	12	0.250
		Metrict	670.6	35	0.920	0.085	0.063	0.006	10.5	5	0.063
		Scalar	651.7	40	0.923	0.078	0.063	0.003	3.0	5	0.704
	Work in other institution ^a^	Configural	679.1	18	0.916	0.122	0.063	–	–	–	–
		Thresholds	759.6	30	0.908	0.099	0.063	−0.009	27.9	12	0.006
		Metrict	709.7	35	0.915	0.088	0.064	0.007	9.3	5	0.099
		Scalar	970.3	40	0.882	0.097	0.064	−0.032	153.3	5	0.000
	Work-related illness	Configural	632.2	18	0.920	0.117	0.062	–	–	–	–
		Thresholds	687.6	30	0.914	0.094	0.062	−0.006	16.4	12	0.172
		Metrict	646.5	35	0.920	0.084	0.063	0.006	13.5	5	0.019
		Scalar	729.0	40	0.911	0.830	0.064	−0.009	49.0	5	0.000
	Chronic disease	Configural	664.7	18	0.918	0.120	0.063	–	–	–	–
		Thresholds	727.3	30	0.911	0.097	0.063	−0.006	19.5	12	0.077
		Metrict	661.4	35	0.920	0.085	0.063	0.009	6.7	5	0.243
		Scalar	651.3	40	0.922	0.078	0.063	0.002	9.4	5	0.095
	Time working ^a^	Configural	754.3	36	0.910	0.127	0.066	–	–	–	–
		Thresholds	825.7	72	0.906	0.092	0.066	−0.004	45.7	36	0.130
		Metrict	753.9	87	0.917	0.079	0.068	0.011	20.4	15	0.016
		Scalar	814.1	102	0.911	0.075	0.068	−0.006	44.9	15	0.000
Health Services Management Satisfaction Scale	Sex	Configural	1446.1	40	0.977	0.119	0.042	–	–	–	–
		Thresholds	1299.7	56	0.980	0.095	0.042	0.003	24.1	16	0.087
		Metrict	1267.4	63	0.980	0.088	0.043	0.001	27.0	7	0.000
		Scalar	1317.7	70	0.980	0.085	0.043	−0.001	50.8	7	0.000
	Age	Configural	1542.4	80	0.976	0.121	0.044	–	–	–	–
		Thresholds	1364.9	128	0.980	0.088	0.044	0.004	84.2	48	0.001
		Metrict	1257.1	149	0.982	0.077	0.044	0.002	24.5	21	0.269
		Scalar	1325.6	170	0.981	0.074	0.044	−0.001	51.2	21	0.000
	Civil status	Configural	1445.7	40	0.977	0.119	0.042	–	–	–	–
		Thresholds	1289.1	56	0.980	0.094	0.042	0.003	12.7	16	0.694
		Metrict	1194.2	63	0.982	0.085	0.043	0.002	14.1	7	0.050
		Scalar	1172.5	70	0.982	0.080	0.043	0.000	11.0	7	0.139
	With specialty	Configural	1456.5	60	0.977	0.119	0.043	–	–	–	–
		Thresholds	1199.4	92	0.982	0.085	0.043	0.005	36.5	32	0.267
		Metrict	1111.8	106	0.984	0.076	0.043	0.002	15.5	14	0.343
		Scalar	1168.4	120	0.983	0.073	0.043	− 0.001	41.8	14	0.000
	Work in other institution	Configural	1483.5	40	0.976	0.121	0.044	–	–	–	–
		Thresholds	1377.8	56	0.978	0.097	0.043	0.002	39.1	16	0.001
		Metrict	1319.4	53	0.979	0.900	0.043	0.001	21.0	7	0.004
		Scalar	1327.1	70	0.979	0.085	0.043	0.000	28.4	7	0.000
	Work-related illness	Configural	1446.0	40	0.977	0.119	0.043	–	–	–	–
		Thresholds	1289.1	56	0.980	0.094	0.043	0.003	16.2	16	0.442
		Metrict	1154.5	53	0.982	0.083	0.043	0.002	9.9	7	0.197
		Scalar	1141.3	70	0.982	0.078	0.043	0.000	14.5	7	0.043
	Chronic disease	Configural	1448.5	40	0.977	0.119	0.043	–	–	–	–
		Thresholds	1278.8	56	0.980	0.094	0.043	0.003	18.2	16	0.311
		Metrict	1163.0	53	0.982	0.084	0.043	0.002	7.2	7	0.410
		Scalar	1129.2	70	0.983	0.078	0.043	0.001	6.9	7	0.434
	Time working	Configural	1480.5	80	0.978	0.119	0.043	–	–	–	–
		Thresholds	1311.4	128	0.981	0.086	0.043	0.003	83.6	48	0.001
		Metrict	1216.8	149	0.983	0.076	0.044	0.002	21.9	21	0.408
		Scalar	1345.0	170	0.981	0.075	0.044	−0.002	79.3	21	0.000
Satisfaction scale on the working conditions of the health center	Sex	Configural	1880.1	86	0.946	0.092	0.055	–	–	–	–
		Thresholds	1991.2	108	0.944	0.084	0.055	−0.003	30.0	22	0.119
		Metrict	1905.0	117	0.946	0.078	0.055	0.003	31.7	9	0.000
		Scalar	1979.6	126	0.945	0.077	0.055	−0.002	72.9	9	0.000
	Age	Configural	1850.8	172	0.949	0.089	0.055	–	–	–	–
		Thresholds	2030.1	238	0.945	0.078	0.055	−0.003	101.7	66	0.003
		Metrict	1794.9	265	0.953	0.068	0.055	0.008	26.4	27	0.499
		Scalar	1937.3	292	0.950	0.067	0.055	−0.004	89.8	27	0.000
	Civil status	Configural	1784.8	86	0.949	0.089	0.054	–	–	–	–
		Thresholds	1889.2	108	0.946	0.081	0.054	−0.002	26.4	22	0.237
		Metrict	1741.4	117	0.951	0.075	0.054	0.005	8.1	9	0.523
		Scalar	1733.6	126	0.952	0.072	0.054	0.001	23.5	9	0.005
	With specialty	Configural	1829.2	129	0.949	0.089	0.054	–	–	–	–
		Thresholds	1922.9	173	0.947	0.078	0.054	−0.001	47.8	44	0.322
		Metrict	1763.5	191	0.953	0.070	0.055	0.005	32.5	18	0.019
		Scalar	2047.4	209	0.945	0.073	0.055	−0.008	138.7	18	0.000
	Work in other institution	Configural	1775.6	86	0.949	0.089	0.053	–	–	–	–
		Thresholds	1886.9	108	0.946	0.081	0.053	−0.003	31.0	22	0.097
		Metrict	1778.1	117	0.950	0.076	0.054	0.004	18.2	9	0.033
		Scalar	1779.8	126	0.950	0.073	0.054	0.000	27.1	9	0.001
	Work-related illness	Configural	1781.1	86	0.949	0.089	0.054	–	–	–	–
		Thresholds	1889.6	108	0.947	0.081	0.054	−0.003	35.2	22	0.035
		Metrict	1732.0	117	0.952	0.075	0.055	0.005	12.7	9	0.175
		Scalar	1739.8	126	0.952	0.072	0.055	0.000	28.6	9	0.001
	Chronic disease	Configural	1768.3	86	0.950	0.089	0.053	–	–	–	–
		Thresholds	1851.7	108	0.948	0.081	0.053	−0.002	22.3	22	0.444
		Metrict	1723.9	117	0.952	0.074	0.053	0.004	16.0	9	0.067
		Scalar	1708.6	126	0.953	0.071	0.054	0.001	18.9	9	0.026
	Time working	Configural	1894.6	172	0.949	0.090	0.056	–	–	–	–
		Thresholds	2063.4	238	0.946	0.079	0.056	−0.003	99.3	66	0.005
		Metrict	1934.3	265	0.950	0.071	0.057	0.005	54.5	27	0.001
		Scalar	2123.5	292	0.946	0.071	0.057	−0.005	120.9	27	0.000

X^2^-Robust = Chi squared Robust. *gl* = Degrees of freedom. *CFI* Comparative fit index. *TLI* Tucker-Lewis index. *RMSEA* Root mean square error of approximation. *SRMR* Standardized root mean square residual. *ΔCFI* Variation of the Comparative-Fit-Index. *DIFFTEST* ANOVA difference test. ^a^ The measurement invariance is not met between the groups

#### Health services management satisfaction scale and satisfaction scale on the working conditions of the health center

Subsequently, we made comparisons between these groups using each of these scales. In both scales, invariance was reached according to sex, age groups, marital status, speciality, working in another institution, time working, work-related disease, and chronic disease (see Table 4). However, it was impossible to evaluate the invariance according to the type of organization and the monthly income since they did not meet the required assumptions.

### Reliability

The *Satisfaction scale on general professional activity* (α = 0.70; ω = 0.70; 6 items) and the *Health services management satisfaction scale* (α = 0.90; ω = 0.90; 3 items) presented adequate internal consistency values. In addition, the *satisfaction scale on the working conditions of the health center* presented adequate values of internal consistency for both the individual working conditions dimension (α = 0.81, ω = 0.81, eight items) and the structural working conditions dimension (α = 0.81, ω = 0.82, three items).

### Satisfaction levels

These exploratory findings showed that the item related to the satisfaction about working conditions with the highest satisfaction level was “satisfaction about the relationship with coworkers” 87.1% of satisfied physicians, and the item with the lowest satisfaction level was “satisfaction about the instruments and equipment to treat patients” had 31.9%. The item related to satisfaction with the professional activity with the highest satisfaction level was “dealing with patients during the consultation (Doctor-patient relationship)” with 94.1% of satisfied physicians, and the item on “risks associated with the profession” has the lowest level of satisfaction 37.9%. Finally, the item related to satisfaction with health service management with the highest satisfaction level was “work scheduling” with 51.9% of satisfied physicians, and the item on “budget management” has the lowest satisfaction level of 21.8% (see Supplement 3).

## Discussion

### Main findings

The set of three independents scales instruments proved a solid factorial structure and measurement invariance, making it possible for group comparison. Stability was achieved with adequate internal consistency coefficients. Based on our findings, these instruments are suitable for measuring job satisfaction in physicians who work in outpatient clinic in the Peruvian health system, as our data is nationally representative. These could become a valuable tool in evaluating different aspects of job satisfaction in physicians, could guide decision-making in human resources and health services research. We consider that these scales can assess different aspects of physicians’ job satisfaction or independently assess specific areas of job satisfaction.

### Contrasting findings with literature

Between 2000 and 2017, a systematic review identified 61 studies evaluating job satisfaction in physicians had been carried out in Europe, 26 different instruments were used to assess it [38]. Moreover, 31% of the studies included development of their own instruments to assess the job satisfaction [38]. The significant heterogeneity of the instruments used in the European context could be related to differences in how these healthcare systems are organized and function, since using a single instrument could lead to biased conclusions. On the other hand, in Latin America, there is no data reporting which are the most frequently used instruments to assess job satisfaction. However, some studies in this region have adopted various instruments to assess it, the Copenhagen Psychosocial Questionnaire [39] or the Warr–Cook–Wall Job Satisfaction Scale are some of the examples [40]. However, these studies had small sample sizes, which cannot represent nationally, and the selected instruments were initially designed in different healthcare contexts in the European region.

In Latin American region, data on this topic is limited, and a great variety of scales developed elsewhere need further analysis and testing. However, the three scales presented in our study have been created considering the peculiarities of a middle-income country in the Latin American region like Peru, and reported adequate evidence of validity and reliability. For example, the consideration that there are primary care centers where water, drainage, and light may not be available permanently (item c2p81_11). And many health professionals tend to work in several institutions at the same time (items c2p82_3), or on how rotations or changes in opening hours are organized (item c2p83_5). Therefore, these scales can be used as a set of tools to evaluate the different aspects of job satisfaction in physicians in Peru and other Spanish-speaking countries with similar healthcare contexts.

#### Factor analysis

We found the evidence of the internal structure of the scales resulting from exploratory and CFA. Our analyses indicate that both the professional activity satisfaction scale and the health center management scale are one-dimension scales (i.e., all items can be added up to obtain an overall score) [24]. The satisfaction scale on the working conditions was a two-dimension scale (individual and structural dimensions), so it is possible to obtain an independent score for each dimension [24].

Our three instruments allow us to collect the information on different aspects of job satisfaction in physicians, considering the peculiarities of the health system in a middle-income country. The general professional activity scale evaluates the availability of physicians to work as a care staff in other institutions (item c2p82_3), since in Peru, around half of the physicians work in more than one institution. On the other hand, the health services management scale assess satisfaction with how the rotating shifts are managed (item c2p83_5) and drug management (item c2p83_2). We have not identified any other scale in the literature that assesses satisfaction with how health centers are managed. And the working conditions scale allows us to assess the satisfaction of the position in the institution (item c2p81_6), and with the essential services such as water or drainage (item c2p81_11).

Moreover, certain items of the scales (professional activity, health center management, individual and structural working conditions) are theoretically similar to other psychometric scales reported in the past. For example, the 4CornerSAT questionnaire used to measure physicians’ career satisfaction has four dimensions analogous similar to the ones we have identified in this study (personal, professional, performance, and inherent) [41, 42].

#### Measurement invariance

The number of studies that evaluated the measurement of invariance of job satisfaction scales is limited, very few instruments have enough evidence to justify the comparisons between groups. However, the comparison between groups is widespread, even when there is no enough evidence to carry out this analysis. It could end in biased results if invariance is violated [29]. One study identified that invariance was achieved by comparing the outcomes of physicians and nurses from 14 European countries, suggesting that cultural factors allow different organizational variables to be assessed in these professionals over time [43]. Our study tested the measurement of invariance of the scales, therefore, allows comparisons between different groups such as marital status, whether they have occupational or chronic diseases. In addition, for the working conditions and health center management scales, further comparisons can be made between men and women, age groups, speciality, working in another institution, and service times. Based on our findings, all these comparisons are free of measurement biases [29].

#### Reliability

We found stability in the scores of three scales. The advantage of our scales compared to others is that they are reasonably short and report adequate levels of internal consistency. It is relevant since many scales like the Warr–Cook–Wall job satisfaction scale, 38 provide little variability and require many items to achieve stable values [26]. We also reported the alpha index mainly due to its widespread use in internal consistency reliability studies; however, its calculus requires certain assumptions: tau equivalence, uncorrelated errors and multivariate normality [44]. In addition, we found differences between the reliability indices of the scales. The *Satisfaction with General Professional Activity Scale* (six items) has reliability coefficients close to the lower limit (α and ω = 0.70). Meanwhile, the *Satisfaction with Health Services Management Scale* (eight items) has high internal consistency indices (α and ω = 0.90). This difference may be because the consistency coefficients are sensitive to the number of items [26]. Since one of the instruments has more items than the other, this could partially explain the difference between the internal consistency indices between the scales.

### Strengths and limitations

One of the strengths of our study was the representativeness of the results at the country level, specifically in Peru, allowing us to test the scales in different outpatient settings. Moreover, ENSUSALUD 2016 has an advanced quality control and real-time monitoring of data collection. However, four significant limitations were recognized. First, despite having the evidence of internal structure for the three instruments, our scales lacked a cut-off period that could determine whether a Peruvian physician was satisfied or not with any of the dimensions evaluated. We found no robust results in the sensitivity and specificity analyses performed for the selected scales. Second, validity and reliability were estimated only for outpatient physicians; therefore, results cannot be generalized to the other health professionals or doctors in other settings such as hospitalization, emergency services or community works; although several doctors usually attend outpatient and work in hospital or emergency services. Third, as this was secondary data analysis, it was impossible to evaluate some variables to better understand the job satisfaction, such as race and mental health illness. Four, we have not access the theoretical review carried out by SUSALUD and the MINSA to formulate the first version of questionnaire 2 of ENSUSALUD. However, when reviewing the literature, we have found a theoretical framework that supports the structure of the scales analyzed.

### Implications in public health, health services management and future research

Healthcare systems worldwide are going through challenging times. Due to the global challenges in demographic, political, economic, and social dimensions of human life, the healthcare field is experiencing an unprecedented changes that threaten the ability of many organizations to promote and protect populations’ health effectively. Many of these healthcare systems struggle to survive too. Healthcare systems need a committed and productive teams to successfully navigate these challenges, including physicians collaborating with organization leaders and the community. Job satisfaction is a relevant dimension to monitor over time, but most measurement scales nowadays are outdated, limited or not culturally translated to other countries and highly diverse territories. In order to support the existence and protective capacity of healthcare systems and promote and maintain population health and wellbeing, job satisfaction needs to be adequately addressed. A future line of research could be to study the properties of job satisfaction instruments in different healthcare systems and identify how each healthcare system influence job satisfaction.

SUSALUD protects and promotes health rights based on the insurance and healthcare provision in the Peruvian health system. Physicians’ job satisfaction became relevant within this competence, although there were no valid and reliable official measurement instruments. In that sense, our study provides novel evidence of the validity and reliability of the ENSUSALUD satisfaction scales to measure job satisfaction among physicians. Having national measures of work satisfaction and other work variables in health professionals represent a valuable tool for decision-makers. In particular, the ENSUSALUD allowed us to have a vision of several critical organizational elements in Peruvian primary care physicians. Our results could contribute to a better measurement of physician satisfaction in Peru. It serves as a basis for making decisions of public policies in human resources of the health area and serves as a source for developing their applicability to Spanish-speaking physicians in other health systems in Latin America and Europe.

We consider that comparing Peruvian regions based on the different areas of job satisfaction (an objective beyond this study’s objective) can be a relevant case for further studies. Also, it is relevant to identify a gold standard of job satisfaction and calculate sensitivity and specificity. In addition, the evaluation of psychometric properties of the three scales in other contexts is a pending task; in that sense, we attach their Spanish (Supplement 1) and English (Supplement 2) versions, respectively. In particular, the English version needs a more comprehensive evaluation.

## Conclusion

The three scales have adequate evidence of validity and reliability and allow job satisfaction evaluation; they could be used to assess the job satisfaction of primary care physicians in Peru. In addition, stakeholders may also use the scales as an indicator of decision-making in the healthcare system.

## Supplementary Information

**Additional file 1: Supplement 1.** *Items of the three instruments (Spanish version) *, the items of each instrument were drafted with their respective Likert scales and instructions for the Spanish version.

**Additional file 2: Supplement 2.** *Preliminary English version of the three instruments*, The items of each instrument were written with their respective Likert scales and instructions for the English preliminary version.

**Additional file 3: Supplement 3.** Percentage of satisfied physicians for each item (*n* = 2137).

## Data Availability

The ENSUSALUD database is publicly available on the SUSALUD website. The link to download the Questionnaire 2 database directly is http://portal.susalud.gob.pe/wp-content/uploads/archivo/base-de-datos/2016/C2_CAPITULOS%20-%20PROFESIONALES%20MEDICOS%20Y%20ENFERMERAS.sav”. ENSUSALUD questionnaire 2 is also available on the website: (http://portal.susalud.gob.pe/blog/encuestas-de-satisfaccion-a-nivel-nacional-ensusalud-2016/).

## Abbreviations

AFPHS: Armed Forces and Police Health Services
CFA: Confirmatory factor analysis
EFA: Exploratory factor analysis
ENSUSALUD: National Survey of Satisfaction of Users in Health
EsSalud: Peruvian Social Security
INEI: Peruvian National Institute of Statistics
KMO: Kaiser-Meyer-Olkin
MINSA: Ministry of Health of Peru
PEN: Peruvian Soles (currency)
SUSALUD: National Superintendency of Health
WLSMV: Weighted using least-squares means and variance adjusted
