# Supplementary material for: Development of the set of scales to assess the job satisfaction among physicians in Peru: validity and reliability assessment
Source: BMC Public Health. 2021 Oct 24;21:1932. doi: 10.1186/s12889-021-11964-6 (PMC8543768; doi:10.1186/s12889-021-11964-6)
Supplement: Supplementary file 1 — Additional file 1: Supplement 1. *Items of the three instruments (Spanish version) *, the items of each instrument were drafted with their respective Likert scales and instructions for the Spanish version. [file 12889_2021_11964_MOESM1_ESM.docx]

**Supplement 1.** Items of the three instruments (Spanish version).

**SPANISH VERSION**

**Question 81: Satisfaction scale on the working conditions of the health center**

***Respecto a su trabajo en este establecimiento de salud, ¿cómo calificaría su nivel de satisfacción en cuanto a:***

| **5** | **4** | **3** | **2** | **1** |
| --- | --- | --- | --- | --- |
| Muy satisfecho/a | Satisfecho/a | Ni insatisfecho/a Insatisfecho/a | Insatisfecho/a | Muy insatisfecho/a |

| Código | Ítems | 5 | 4 | 3 | 2 | 1 |
| --- | --- | --- | --- | --- | --- | --- |
| c2p81_1 | Posibilidades de promoción o ascenso? |  |  |  |  |  |
| c2p81_2* | Orden de los servicios y organización laboral? * |  |  |  |  |  |
| c2p81_3 | La valoración de su trabajo? |  |  |  |  |  |
| c2p81_4 | El tipo de labor que realiza? |  |  |  |  |  |
| c2p81_5 | La carga laboral que realiza? |  |  |  |  |  |
| c2p81_6 | Posición en su institución y participación en las decisiones de gestión de su servicio? |  |  |  |  |  |
| c2p81_7* | Honorarios o sueldos recibidos* |  |  |  |  |  |
| c2p81_8 | Horario o jornada de trabajo? |  |  |  |  |  |
| c2p81_9 | La relación con sus compañeros de trabajo? |  |  |  |  |  |
| c2p81_10* | Oportunidades de capacitación/actualización? * |  |  |  |  |  |
| c2p81_11 | La infraestructura e instalaciones de servicios (agua, desagüe, luz, oxígeno, etc.)? |  |  |  |  |  |
| c2p81_12 | El instrumental y equipamiento para atender a los pacientes? |  |  |  |  |  |
| c2p81_13 | La relación con sus jefes o superiores? |  |  |  |  |  |
| c2p81_14 | Las condiciones de higiene y bioseguridad? |  |  |  |  |  |
| c2p81_15* | El llenado de registro, partes, órdenes o formatos? (Excluye historia clínica) * |  |  |  |  |  |
| c2p81_16* | El respeto y consideración de sus pacientes?* |  |  |  |  |  |

Nota: * ítems que son eliminados en el análisis final.

**Question 82: Satisfaction scale on general professional activity**

***En relación a su actividad profesional en general, ¿cómo calificaría usted su nivel de satisfacción, respecto a:***

| **5** | **4** | **3** | **2** | **1** |
| --- | --- | --- | --- | --- |
| Muy satisfecho/a | Satisfecho/a | Ni insatisfecho/a Insatisfecho/a | Insatisfecho/a | Muy insatisfecho/a |

| Código | Ítems | 5 | 4 | 3 | 2 | 1 |
| --- | --- | --- | --- | --- | --- | --- |
| c2p82_1 | La relación médico paciente durante la consulta? |  |  |  |  |  |
| c2p82_2 | Su expectativa en satisfacer las necesidades de sus pacientes? |  |  |  |  |  |
| c2p82_3 | Su disponibilidad para realizar ejercicio profesional asistencial en otras instituciones? |  |  |  |  |  |
| c2p82_4 | Los logros obtenidos en su carrera? |  |  |  |  |  |
| c2p82_5 | Impacto en su vida personal o familiar por la carga laboral asociada a su profesión? |  |  |  |  |  |
| c2p82_6 | Los riesgos asociados a su actividad profesional? |  |  |  |  |  |

**Question 83: Health Services Management Satisfaction Scale**

***En relación al equipo de gestión de su establecimiento, ¿cómo calificaría usted su nivel de satisfacción, respecto a:***

| **5** | **4** | **3** | **2** | **1** |
| --- | --- | --- | --- | --- |
| Muy satisfecho/a | Satisfecho/a | Ni insatisfecho/a Insatisfecho/a | Insatisfecho/a | Muy insatisfecho/a |

| Código | Ítems | 5 | 4 | 3 | 2 | 1 |
| --- | --- | --- | --- | --- | --- | --- |
| c2p83_1 | Manejo de presupuesto? |  |  |  |  |  |
| c2p83_2 | Gestión de medicamentos - farmacias? |  |  |  |  |  |
| c2p83_3 | Organización de los servicios? |  |  |  |  |  |
| c2p83_4 | Gestión de los recursos humanos? |  |  |  |  |  |
| c2p83_5 | Programación de turnos? |  |  |  |  |  |
| c2p83_6 | Atención al usuario? |  |  |  |  |  |
| c2p83_7 | Prevención de infecciones intrahospitalarias / eventos adversos? |  |  |  |  |  |
| c2p83_8 | Capacidad de gestión / trabajo? |  |  |  |  |  |
