# Supplementary material for: Development of the set of scales to assess the job satisfaction among physicians in Peru: validity and reliability assessment
Source: BMC Public Health. 2021 Oct 24;21:1932. doi: 10.1186/s12889-021-11964-6 (PMC8543768; doi:10.1186/s12889-021-11964-6)
Supplement: Supplementary file 2 — Additional file 2: Supplement 2. *Preliminary English version of the three instruments*, The items of each instrument were written with their respective Likert scales and instructions for the English preliminary version. [file 12889_2021_11964_MOESM2_ESM.docx]

**Supplement 2.** Preliminary English version of the three scales evaluated

**ENGLISH VERSION**

**Question 81: Satisfaction scale on the working conditions of the health center**

***Regarding your work in this health facility, how would you rate your satisfaction level in terms of the:***

| **5** | **4** | **3** | **2** | **1** |
| --- | --- | --- | --- | --- |
| Very satisfied | Satisfied | Neither unsatisfied nor dissatisfied | Unsatisfied | Very unsatisfied |

| Code | Items | 5 | 4 | 3 | 2 | 1 |
| --- | --- | --- | --- | --- | --- | --- |
| c2p81_1 | Opportunities for advancement or promotion |  |  |  |  |  |
| c2p81_2* | Order of services and labor organization* |  |  |  |  |  |
| c2p81_3 | Appreciation of work by coworkers |  |  |  |  |  |
| c2p81_4 | Nature of work carried out |  |  |  |  |  |
| c2p81_5 | Workload |  |  |  |  |  |
| c2p81_6 | Position in the institution and participation in management decisions of the medical service. |  |  |  |  |  |
| c2p81_7* | Salary or remuneration* |  |  |  |  |  |
| c2p81_8 | Working hours |  |  |  |  |  |
| c2p81_9 | Relationship with coworkers |  |  |  |  |  |
| c2p81_10* | Skills training/updating opportunities* |  |  |  |  |  |
| c2p81_11 | Physical plant and service facilities (water suply, drainage, power supply, ventilation, etc.) |  |  |  |  |  |
| c2p81_12 | Instruments and equipment to treat patients |  |  |  |  |  |
| c2p81_13 | Relationship with bosses and superiors |  |  |  |  |  |
| c2p81_14 | Hygienic and biosecure conditions of workplace |  |  |  |  |  |
| c2p81_15* | Filling out forms, medical parts, orders and formats (excluding medical records) * |  |  |  |  |  |
| c2p81_16* | Respect and consideration from patients* |  |  |  |  |  |

Note: * items that are eliminated in the final analysis.

**Question 82: Satisfaction scale on general professional activity**

***Regarding your general professional activity, how would you rate your satisfaction level in terms of the:***

| **5** | **4** | **3** | **2** | **1** |
| --- | --- | --- | --- | --- |
| Very satisfied | Satisfied | Neither unsatisfied nor dissatisfied | Unsatisfied | Very unsatisfied |

| Code | Items | 5 | 4 | 3 | 2 | 1 |
| --- | --- | --- | --- | --- | --- | --- |
| c2p82_1 | Dealing with patients during consultation (Doctor-patient relationship) |  |  |  |  |  |
| c2p82_2 | Expectation to meet the needs of your patients |  |  |  |  |  |
| c2p82_3 | Willingness to extend professional care in other institutions |  |  |  |  |  |
| c2p82_4 | Career achievements |  |  |  |  |  |
| c2p82_5 | Impact of workload on your personal and/or family life |  |  |  |  |  |
| c2p82_6 | Risks associated with the profession |  |  |  |  |  |

**Question 83: Health Services Management Satisfaction Scale**

***Regarding the management in this health facility, how would you rate your satisfaction level in terms of the:***

| **5** | **4** | **3** | **2** | **1** |
| --- | --- | --- | --- | --- |
| Very satisfied | Satisfied | Neither unsatisfied nor dissatisfied | Unsatisfied | Very unsatisfied |

| Code | Items | 5 | 4 | 3 | 2 | 1 |
| --- | --- | --- | --- | --- | --- | --- |
| c2p83_1 | Budget management |  |  |  |  |  |
| c2p83_2 | Drug/Pharmacy management |  |  |  |  |  |
| c2p83_3 | Organization of services |  |  |  |  |  |
| c2p83_4 | Human resources management |  |  |  |  |  |
| c2p83_5 | Work scheduling |  |  |  |  |  |
| c2p83_6 | User support |  |  |  |  |  |
| c2p83_7 | Prevention of nosocomial infections/adverse events |  |  |  |  |  |
| c2p83_8 | Management/work capacity |  |  |  |  |  |
