# Supplementary material for: Development of the set of scales to assess the job satisfaction among physicians in Peru: validity and reliability assessment
Source: BMC Public Health. 2021 Oct 24;21:1932. doi: 10.1186/s12889-021-11964-6 (PMC8543768; doi:10.1186/s12889-021-11964-6)
Supplement: Supplementary file 3 — Additional file 3: Supplement 3. Percentage of satisfied physicians for each item (n = 2137). [file 12889_2021_11964_MOESM3_ESM.docx]

**Supplement 3.** Percentage of satisfied physicians for each item (n=2,137)

| **Scale** | **Code** | **Items** | **% satisfied** | **% dissatisfied** |
| --- | --- | --- | --- | --- |
| Satisfaction scale on the working conditions of the health center | c2p81_1 | Opportunities for advancement or promotion. | 39.9% | 60.2% |
|  | c2p81_3 | Appreciation of work by coworkers. | 63.5% | 36.6% |
|  | c2p81_4 | Nature of work carried out. | 82.8% | 17.2% |
|  | c2p81_5 | Workload. | 65.2% | 34.8% |
|  | c2p81_6 | Position in the institution and participation in management decisions of the medical service. | 45.7% | 54.3% |
|  | c2p81_8 | Working hours. | 59.7% | 40.4% |
|  | c2p81_9 | Relationship with coworkers. | 87.1% | 12.9% |
|  | c2p81_11 | Physical plant and service facilities (water suply, drainage, power supply, ventilation, etc.) | 37.9% | 62.1% |
|  | c2p81_12 | Instruments and equipment to treat patients. | 31.4% | 68.6% |
|  | c2p81_13 | Relationship with bosses and superiors. | 69.2% | 30.8% |
|  | c2p81_14 | Hygienic and biosecure conditions of workplace. | 46.2% | 53.8% |
| Satisfaction scale on general professional activity | c2p82_1 | Dealing with patients during consultation (Doctor-patient relationship). | 94.1% | 5.9% |
|  | c2p82_2 | Expectation to meet the needs of your patients. | 84.0% | 16.0% |
|  | c2p82_3 | Willingness to extend professional care in other institutions. | 59.3% | 40.7% |
|  | c2p82_4 | Career achievements. | 82.2% | 17.8% |
|  | c2p82_5 | Impact of workload on your personal and/or family life. | 52.3% | 47.7% |
|  | c2p82_6 | Risks associated with the profession. | 37.9% | 62.2% |
| Health Services Management Satisfaction Scale | c2p83_1 | Budget management. | 21.8% | 78.2% |
|  | c2p83_2 | Drug/Pharmacy management. | 22.8% | 77.2% |
|  | c2p83_3 | Organization of services. | 34.2% | 65.8% |
|  | c2p83_4 | Human resources management. | 27.0% | 73.0% |
|  | c2p83_5 | Work scheduling. | 51.9% | 48.1% |
|  | c2p83_6 | User support. | 49.1% | 50.9% |
|  | c2p83_7 | Prevention of nosocomial infections/adverse events. | 43.6% | 56.4% |
|  | c2p83_8 | Management/work capacity. | 37.8% | 62.2% |
